# A pyroptosis-related gene signature for prognostic and immunological evaluation in breast cancer

**DOI:** 10.3389/fonc.2022.964508

**Published:** 2022-12-23

**Authors:** Yue Zhong, Fu Peng, Xiaoru Luo, Xuan Wang, Bowen Yang, Xinglinzi Tang, Zheng Xu, Linlin Ren, Zhiyu Wang, Cheng Peng, Neng Wang

**Affiliations:** ^1^ Integrative Medicine Research Center, School of Basic Medical Sciences, Guangzhou University of Chinese Medicine, Guangzhou, Guangdong, China; ^2^ State Key Laboratory of Southwestern Chinese Medicine Resources, Chengdu University of Traditional Chinese Medicine, Chengdu, Sichuan, China; ^3^ Key Laboratory of Drug-Targeting and Drug Delivery System of the Education Ministry and Sichuan Province, West China School of Pharmacy, Sichuan University, Chengdu, China; ^4^ State Key Laboratory of Dampness Syndrome of Chinese Medicine, The Second Affiliated Hospital of Guangzhou University of Chinese Medicine, Guangzhou, Guangdong, China; ^5^ Guangdong Provincial Key Laboratory of Clinical Research on Traditional Chinese Medicine Syndrome, Guangdong Provincial Academy of Chinese Medical Sciences, Guangdong Provincial Hospital of Chinese Medicine, Guangzhou, Guangdong, China; ^6^ Guangdong-Hong Kong-Macau Joint Lab on Chinese Medicine and Immune Disease Research, Guangzhou University of Chinese Medicine, Guangzhou, Guangdong, China

**Keywords:** breast cancer, pyroptosis, 4-gene signature, survival status, immunological landscape

## Abstract

**Purpose:**

Pyroptosis exerts an undesirable impact on the clinical outcome of breast cancer. Since any single gene is insufficient to be an appropriate marker for pyroptosis, our aim is to develop a pyroptosis-related gene (PRG) signature to predict the survival status and immunological landscape for breast cancer patients.

**Methods:**

The information of breast cancer patients was retrieved from The Cancer Genome Atlas (TCGA) and the Gene Expression Omnibus (GEO) databases. Quantitative real-time polymerase chain reaction (qRT-PCR) was performed to verify the gene expressions of this signature in breast cancer. Its prognostic value was evaluated by univariate Cox analysis, least absolute shrinkage and selection operator (LASSO) regression analysis, receiver operating characteristics (ROCs), univariate/multivariate analysis, and nomogram. Analyses of Gene Ontology (GO) and Kyoto Encyclopedia of Genes and Genomes (KEGG) were performed to explore its potential biological function in breast cancer. The potential correlation between this signature and tumor immunity was revealed based on single sample gene set enrichment analysis (ssGSEA), ESTIMATE and CIBERSORT algorithms.

**Results:**

A PRG signature containing GSDMC, GZMB, IL18, and TP63 was created in a TCGA training cohort and validated in two validation GEO cohorts GSE58812 and GSE37751. Compared with a human mammary epithelial cell line MCF-10A, the expression levels of GSDMC, GZMB and IL18 were upregulated, while TP63 was found with lower expression level in breast cancer cells SK-BR-3, BT-549, MCF-7, and MDA-MB-231 using RT-qPCR assay. Based on univariate and multivariate Cox models, ROC curve, nomogram as well as calibration curve, it was revealed that this signature with high-risk score could independently predict poor clinical outcomes in breast cancer. Enrichment analyses demonstrated that the involved mechanism was tightly linked to immune-related processes. SsGSEA, ESTIMATE and CIBERSORT algorithms further pointed out that the established model might exert an impact on immune cell abundance, immune cell types and immune-checkpoint markers. Furthermore, individuals with breast cancer responded differently to these therapeutic agents based on this signature.

**Conclusions:**

Our data suggested that this PRG signature with high risk was tightly associated with impaired immune function, possibly resulting in an unfavorable outcome for breast cancer patients.

## 1 Introduction

Breast cancer belongs to one of the most common cancer diseases and ranks first or second in mortality rates among women worldwide, with approximately 2,260,000 increasing cases and almost 685,000 deaths according to the GLOBOCAN 2020 estimation (1). At present, a variety of anti-breast cancer treatments are available, including surgical operations, chemotherapeutic options, radiotherapeutic plans, hormone-based strategies, targeted therapies and others. Appropriate therapeutic measures are taken based on different breast cancer subtypes, such as HR+/ERBB2− (seven-tenths of the total), ERBB2+ (one-fifth of the total) as well as triple-negative (one-tenth of the total). The median overall survival (OS) for the first two subtypes is 5 years in comparison with approximately 1 year for the triple-negative phenotype. Accordingly, the main goal for treating the first two subtypes is eradication of local breast tumors/regional lymph nodes to prevent increasing risks of recurrence, while the therapeutic value in treating triple-negative phenotype is to prolong life span and alleviate patients’ suffering (2). Although more than 90% breast cancer patients are initially diagnosed as non-metastatic phenotypes, 20–50% of them eventually develop into advanced stages or distant recurrent phenotypes of breast cancer (3). In addition, tumor progression is highly dependent on the tumor niche or tumor microenvironment (TME). Immune dysregulation always leads to ineffectiveness and even multidrug resistance of clinical agents in cancer modality therapies (4). TME-associated oncogenes and/or tumor suppressor genes have potential value in determining tumor typing, gene sets, and pathways as well as phenotype modeling for research into breast cancer (5). Hence, it is urgent and essential to develop and validate a prognostic model to predict OS and immunological landscape in patients with breast cancer.

Pyroptosis is a certain kind of programmable cell death mediated by inflammasomes. It has been characterized by the formation of inflammasomes, the activation of caspase and gasdermin, as well as the release of pro-inflammatory cytokines (5). In the canonical pathway, pyrolytic cells appear light swelling with many bubble-like inflammasomes. The inflammasomes then join together and recruit caspase-1 to activate GSDMD within its N-terminal domain, and trigger the abnormal expressions of IL1β, IL18, high mobility group box 1 (HMGB1) and others through necrotic membrane pores formed by GSDMD-N. Alternatively, both pyroptosis initiation and GSDMD cleavage are caspase 4, 5, 11-dependent by combining with cellular lipopolysaccharides rather than recruiting inflammasomes in the non-canonical pathway (6). A variety of inflammatory factors are released into TME and blood circulation to promote systemic inflammation during pyroptosis. Therefore, a number of pilot studies have been focused on pyroptosis to comprehend its association with infectious diseases, nervous system disorders, and atherosclerosis-associated diseases (7). Of note, it has also been demonstrated that pyroptosis displays dichotomous behaviors during oncogenesis possibly due to different cell types, genetics and tumor stages. On one hand, tumors with abnormal GSDMD expression, activated inflammasomes, and enhanced pro-inflammatory cytokines belong to a more aggressive phenotype by maintaining immunosuppressive cells, facilitating stromal cell transformation from epithelial cells, and up-regulating matrix metalloproteinases for extracellular matrix remodeling (6). On the other hand, pyroptosis exerts anti-tumor function. For instance, pyroptosis induced by NLRP3-mediated inflammasomes could significantly delay the growth and metastasis of hepatocellular carcinoma (7). Thus, it is necessary to establish diagnostic and prognostic signatures for pyroptosis in order to clarify its significance in breast cancer.

It appears that pyroptosis holds promise as a potential adjuvant in tumor immunotherapy with a good prospect. According to clinical findings, different tumor types respond differently to immunotherapy. Tumors sensitive to immunotherapy are classified as “hot tumors”, while tumors with poor reactivity are called “cold tumors” (8). Thus, different treatment strategies should be adopted for different tumors, and how to transform “cold tumors” into “hot tumors” to improve the curative effect is particularly critical. An exciting research reported the synergistic effect of pyroptosis induction and PD-1 inhibitor could turn the tumor from immune-silent “cold tumors” to “hot tumors” with immune stimulation, suggesting the great potential of this combination (9). Moreover, pyroptosis contributes to tumor inhibition by stimulating anti-tumor immune response. Activating GSDME could promote macrophage phagocytosis and enhance the function of natural killer (NK) cells and CD8+ T cells (10). The study of Tan et al. revealed that DRD2 polarized macrophages to M1 by restricting NF-κB signaling, subsequently resulting in GSDME-induced pyroptosis in breast cancer (11). Overall, investigating the association between pyroptosis and tumor immunity can bring new insights into the prognosis and treatment of breast cancer.

Given the strong correlation between pyroptosis and cancer diseases, it is worth investigating the specific functions of PRGs (12). However, breast cancer-associated PRGs have not been fully explored yet. Herein, this study described a comprehensive analysis of breast cancer-associated PRGs, including their prognostic value, biological function and pathways, immunological characterization, drug sensitivity as well as genomic information. In particular, we developed a PRG-based signature to evaluate its prognostic value for breast cancer patients based on Kaplan-Meier and ROC methods. Subsequently, ssGSEA, ESTIMATE and CIBERSORT algorithms were also applied to clarify the relationship between PRGs and immune cell infiltration in TME, which would provide new targets for breast cancer immunotherapy.

## 2 Materials and methods

### 2.1 Data source and collection

We downloaded the RNA sequencing data of 1109 breast cancer tumor tissues as well as 113 adjacent tissues, and the related clinical information in the TCGA dataset (https://portal.gdc.cancer.gov/repository). Counts value matrix was utilized to screen out differentially expressed genes (DEGs) involved in pyroptosis, while TPM value matrix was used for the rest of analyses. Breast cancer patients with missing OS values or OS ≤30 days were excluded from the analysis to avoid statistical bias. We also used the GEO database (GSE58812 and GSE37751) (https://www.ncbi.nlm.nih.gov/geo/) to retrieve gene expression data and clinical data.

### 2.2 Identification of differentially expressed PRGs

The involved 52 PRGs were obtained from previous studies (13) (**Table S1**). The DEG identification between tumor tissues and adjacent tissues was conducted by the R package “edgeR” (14). Adjusted P-value < 0.05 and |log2 (fold change) | (log2 FC) > 1 were defined as the threshold. Protein-protein interaction (PPI) networks were set up using screened DEGs with the Retrieval of Interacting Genes (STRING) (https://string-db.org/).

### 2.3 Construction of the PRG model in breast cancer

To evaluate the prognostic value of this PRG model, both Cox regression analysis and LASSO regression analysis were utilized to evaluate the relationship between PRGs and the survival status in the TCGA cohort. For Cox regression analysis, genes that met P-value < 0.05 were further analyzed, and 4 survival-related genes were found. A prognostic model was then constructed using R package “glmnet” based on the LASSO regression. Finally, 4 genes were maintained. The penalty parameter (λ) was determined by applying minimum criteria. The risk score was calculated by the following formula:


$$\text{Riskscore}=\sum_{\text{i}=1}^{\text{n}}\;\text{Coef}\;(\text{Xi})*\text{Exp}\;(\text{Xi})$$

For each gene Xi, coef (Xi) represented the coefficient, and Exp (Xi) represented gene expression. Breast cancer patients were classified into two groups (the high- and low-risk groups) based on the median risk score. We used principal component analysis (PCA) with “prcomp” R package and t-distributed stochastic neighbor embedding (t-SNE) test using “Rtsne” R package (15) to analyze two groups’ distribution. Then, Kaplan-Meier analysis was applied to examine the interactions between risk value and the survival time using R packages of “survival” (16) and “survminer” (17), and ROC curve analysis was performed to evaluate their diagnosis index with “timeROC” R package (18).

### 2.4 Validation of PRG signature

To further test and verify the 4-gene signature model based on TCGA, its prediction accuracy was re-evaluated in the GEO pool (GSE58812 and GSE37751). Kaplan-Meier curves implied significant discrepancies between the two risk groups of patients. Then ROC curves analysis was used to confirm the robustness of our PRG prognostic model.

### 2.5 Cell culture

MCF-10A, BT-549, MCF-7 and MDA-MB-231 were purchased from the American Type Culture Collection (ATCC, Manassas, VA, United States). SK-BR-3 was obtained from Jiangsu Kaiji Biotechnology Co., Ltd (Nanjing, Jiangsu, China). The cells were cultured in medium (DMEM for SK-BR-3 and MDA-MB-231 cells; RPMI-1640 for BT-549 and MCF-7 cells) and were added with 10% fetal bovine serum (Gibco, Nork York, NY, United States) as well as 1% penicillin and streptomycin (Gibco, Nork York, NY, United States). Both DMEM and RPMI-1640 were purchased from Gibco company (Gibco, Nork York, NY, United States). MCF-10A was maintained in DMEM/F12 (Gibco, New York, NY, United States) supplemented with 5% horse serum (Hyclone, Logan, UT, United States), 20 ng/ml epidermal growth factor (BD Bioscience, Bedford, MA, United States), 10 μg/ml insulin (Sigma, St. Louis, MO, United States), 0.5 μg/ml hydrocortisone (StemCell Technologies, Vancouver, BC, Canada), 100 ng/ml cholera toxin (Macgene, Beijing, China) and 1% penicillin and streptomycin (Gibco, New York, NY, United States). All these cell lines were kept at 37°C, with a humidified atmosphere of 5% CO2.

### 2.6 RNA isolation and qRT-PCR analysis

The primer sequences of GSDMC, GZMB, IL18, and TP63 were synthesized by WcGene Biotech (Shanghai, China), and β-actin was synthesized by Sangon Biotech (Shanghai, China) (listed in **Table S2**). Total RNA was extracted with an RNA extraction kit (DP419, Tiangen Biotech Beijing Co., Ltd., Beijing, China), followed by reverse transcription reaction using TAKARA reverse transcription kit (RR047A, Takara, Shiga, Japan). Following qRT-PCR analysis was performed with TB Green^®^ Premix Ex Taq™ II (RR820A, Takara, Shiga, Japan) in Bio-Rad CFX96. A comparative Ct method (2^-ΔΔCT^) was used to calculate the expression level of RNA normalized to β-actin.

### 2.7 Independent prognostic analysis

Univariate and multivariate Cox regression was conducted to investigate whether the risk score could be an independent prognostic factor. TCGA was used to obtain the clinical information (age, T stage, N stage, and M stage) of breast patients.

### 2.8 A predictive nomogram construction

A nomogram was established to predict the 1-, 3-, or 5-year survival probability and accuracy performance of the model assessed by calibration curves.

### 2.9 Functional enrichment analysis of PRGs

Patients with breast cancer were stratified by a median risk score into low- and high-risk groups from the TCGA and GEO cohorts. The DEG analysis was performed between two groups using the “limma” package (19). The threshold was set as follows: FDR < 0.05, | log2 FC | > 1. GO (20, 21) and KEGG (22) were performed by using “clusterProfiler” (23), and “ggplot2” (24) R package. The venn diagram was drawn by “VennDiagram” R package (25).

### 2.10 Assessment of immune status and CSC index between two subgroups

SsGSEA was performed to calculate the immune cell infiltration based on “GSVA” R package (26). The R package “ESTIMATE” was utilized to count the scores (immune/stromal/estimate score) and tumor purity in TME (27). R package “CIBERSORT” was used to reveal the intrinsic links between PRG score and immune cells abundance in TCGA (28). Subsequently, we used Spearman’s correlation analysis to analyze the relationship between the risk score and the index of immune cells/cancer stem cells (CSCs). Threshold P-value < 0.05 was considered significant.

### 2.11 Drug sensitivity evaluation

An analysis of half inhibitory concentrations (IC50) of common drugs was performed using “pRRophetic” R software in TCGA (29). And we applied the Wilcoxon signed-rank test to detect IC50 between two subgroups.

### 2.12 Data analysis using cBioPortal

cBioPortal (www.cbioportal.org) is a comprehensive web resource for collection and analysis of cancer genomics data, such as copy number alterations, DNA methylation and so on (30). 2509 samples [Breast Cancer (METABRIC, Nature 2012 & Nat Commun 2016)] were explored, and mRNA expression z-scores (log microarray) were acquired using a z-score threshold of ± 2.0. We also used cBioPortal web platform to analyze the relationship between the TP63 gene expression and its methylation level.

### 2.13 Analysis of common genes between pyroptosis and autophagy

The autophagy-related genes (ARGs) were collected from Human Autophagy Database (http://www.autophagy.lu/) (**Table S3**). An intersection was acquired by ARGs and PRGs. The effects of high and low gene expression on OS were investigated by Kaplan–Meier curves using R packages of “survival” and “survminer” (17).

### 2.14 Statistical analysis

Data analyses were completed by R software (v4.0.1) and SPSS software (version 26). Statistical significance was defined by P < 0.05.

## 3 Results

### 3.1 Identification of pyroptosis-related DEGs in breast cancer

**Figure 1:**
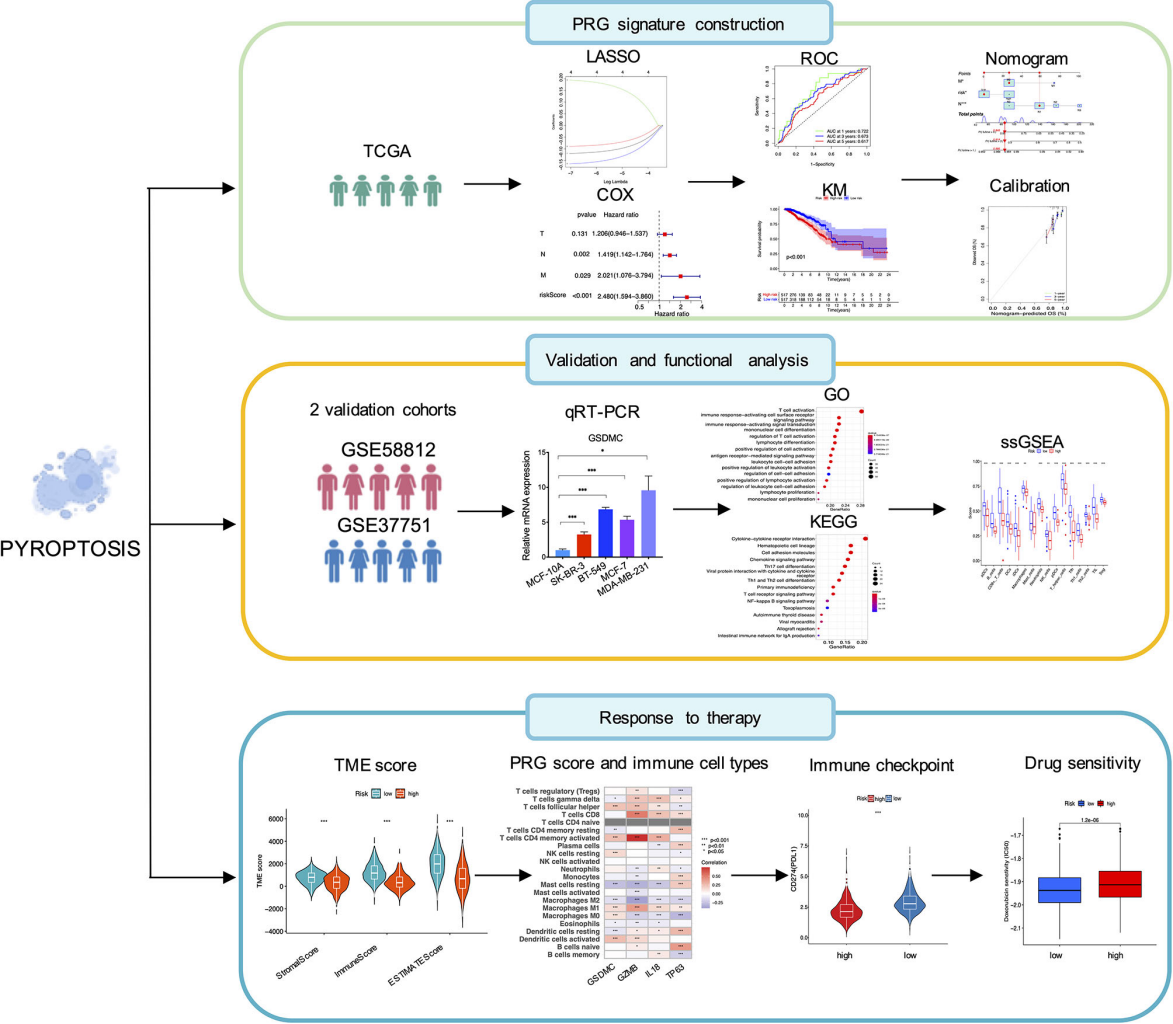
Schematic illustration of the study design, including PRG signature construction, validation and functional analysis and response evaluation to therapies.

**Figure 2:**
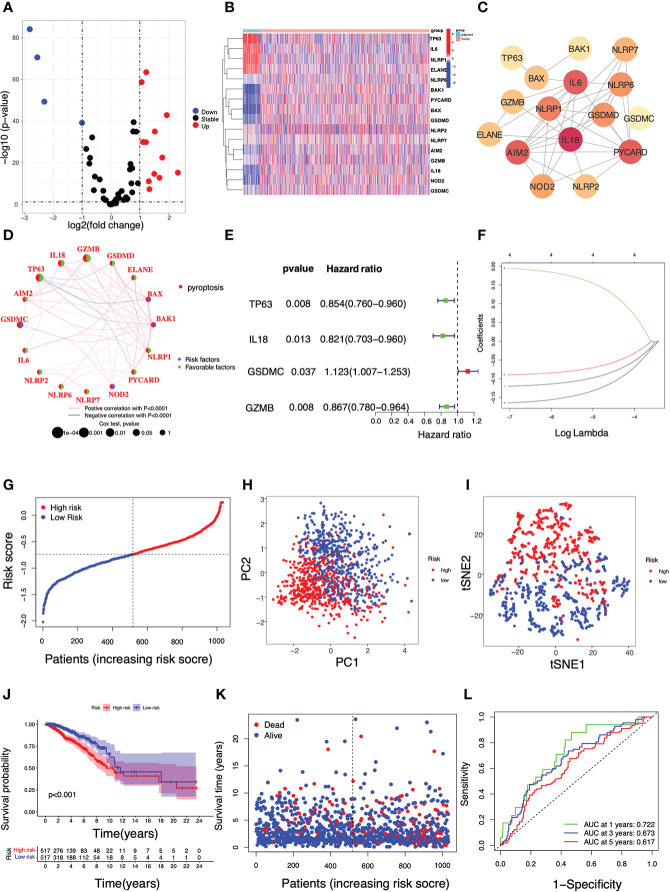
Identification of pyroptosis-related DEGs and construction of a PRG-based prognostic model using the TCGA cohort in breast cancer. **(A)** A volcano plot displaying pyroptosis-related DEGs in breast cancer (P < 0.05, *red*: up-regulated genes; *blue*: down-regulated genes); **(B)** A heatmap of the 16 differential PRG expressions between breast tumors and adjacent tissues (P < 0.05, *blue*: decreased expression; *red*: increased expression); **(C)** A PPI network indicating the intricate interactions of the 16 PRGs (interaction score = 0.4); **(D)** The connection network among PRGs (*Pink*: a positive association; *blue:* a negative association. *Green*: a favorable factor; *purple*: a risk factor). **(E)** Univariate Cox regression analysis of survival-related PRGs (P < 0.05); **(F)** A 4-gene risk model was built based on LASSO regression analysis; **(G)** The breast cancer patient distribution based on the median value of the risk score; **(H)** PCA plot and **(I)** T-SNE analysis in the TCGA cohort; **(J)** Kaplan–Meier curve analysis for the OS of patients with either high or low risk; **(K)** The survival status of each breast cancer patient (*left dotted line*: low-risk; *right dotted lin*e: high-risk); **(L)** ROC analysis curve for the signature of this 4-gene set.

**Figure 1** illustrated the flowchart of this study, including PRG signature construction, validation and functional analysis as well as response evaluation to therapies. We compared expression levels of 52 PRGs with DEGs from 1109 breast tumors and 113 adjacent tissues in TCGA, and identified 16 pyroptosis-related DEGs in breast cancer. Among them, 12 genes were significantly upregulated (BAX, BAK1, PYCARD, NOD2, GSDMD, IL18, AIM2, NLRP7, NLRP6, GSDMC, GZMB, NLRP2), while 4 other genes were obviously downregulated (IL6, TP63, ELANE, NLRP1) according to volcanoes (P < 0.05, **Figure 2A**). The differential PRG expressions were visualized by heatmaps in **Figure 2B**. To further investigate their intricate correlation with each other, a PPI-associated analysis was established in the light of a minimum interaction score of 0.4 (**Figure 2C**), and their interactions were also shown in a pyroptosis-based network (**Figure 2D**).

### 3.2 Construction of a PRG-based prognostic model in TCGA

In the search for the survival-related PRGs, a univariate Cox regression analysis was conducted based on a threshold of P < 0.05. As shown, 4 out of 16 aforementioned DEGs were selected as the appropriate candidates for constructing a pyroptosis-related risk signature. In particular, 3 genes TP63, IL18, and GZMB were shown as protective genes with hazard ratios (HRs) < 1, while GSDMC was regarded as a risk gene with HRs >1 (**Figure 2E**). In the following LASSO regression analysis, a 4-gene risk model was built based on the LASSO optimal λ regression (**Figure 2F**). Accordingly, the risk score was calculated as indicated: Risk score = (−0.120 * TP63 Exp.) + (−0.090 * IL18 Exp.) + (0.194 * GSDMC Exp.) + (−0.164 * GZMB Exp.). Patients with breast cancer were divided into two subgroups *i.e.* one with high risk (n = 517) and the other with low risk (n = 517) distinguished by the median risk score in TCGA (**Figure 2G**). In addition, the PCA and t-SNE analysis showed a high-quality separation between the two subgroups (**Figures 2H, I**). The Kaplan-Meier curve was used to reveal the discrepancies between the two risk groups, and it was found that breast cancer patients with high risk had shorter survival periods than those with low risk (**Figures 2J, K**). ROC analysis was then performed to validate the sensitivity and specificity of this signature, and the areas under curve (AUC) were 0.722 (1-year), 0.673 (3-year) and 0.617 (5-year), respectively (**Figure 2L**).

### 3.3 Signature validation with the GEO cohorts and qRT-PCR assay

**Figure 3:**
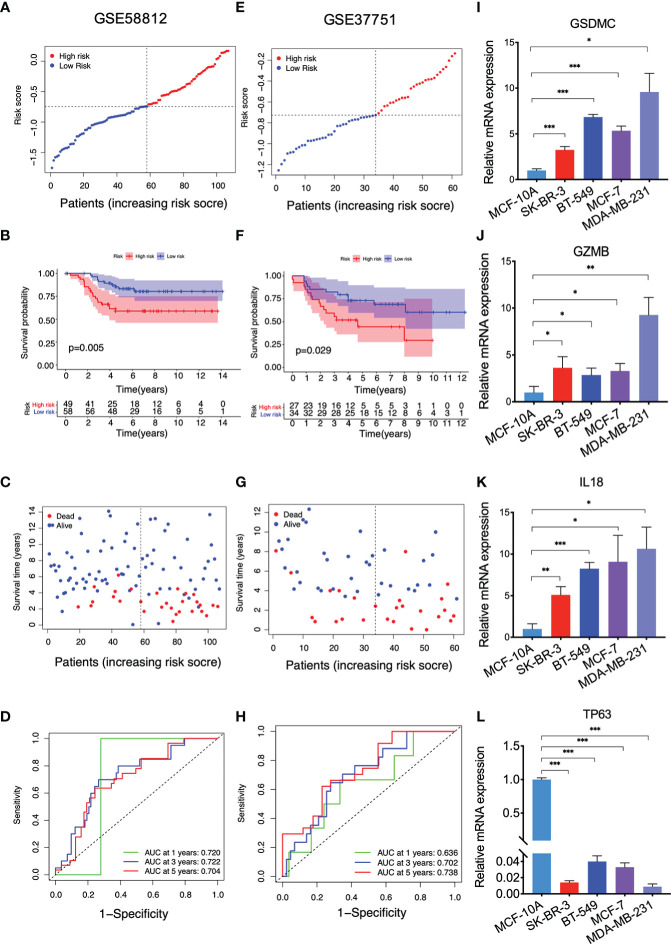
Validation of the PRG model with the GEO cohorts and qRT-PCR results. **(A)** The distribution of breast cancer patients based on the risk score in GSE58812; **(B)** Kaplan-Meier curves for OS in the low and high-risk groups in GSE58812; **(C)** The survival status for each breast cancer patient in GSE58812; **(D)** ROC curve analysis in GSE58812; **(E)** The distribution of breast cancer patients based on the risk score in GSE37751; **(F)** Kaplan-Meier curves for OS in the low and high-risk groups in GSE37751; **(G)** The survival status for each breast cancer patient in GSE37751; **(H)** ROC curve analysis in GSE37751; **(I–L)** qRT-PCR results indicating the expressions of GSDMC, GZMB, IL18, and TP63 in the indicated cell lines, values represented the mean ± SD. n=3, *P<0.05; **P<0.01; ***P<0.001.

Data from GSE58812 and GSE37751 were utilized as two independent validation sets to verify the prognostic value of this model. In GSE58812, 58 breast cancer patients were annotated as the low-risk populations and 49 were characterized by high risk based on the median risk score in the TCGA cohort (**Figure 3A**). In Kaplan–Meier analysis, a significantly lower survival rate was observed in the high-risk group compared to the low-risk group (P = 0.005, **Figure 3B**). The survival status in the indicated groups was presented in **Figure 3C**. The 1-, 3-, and 5-year AUC values were 0.720, 0.722, and 0.704 in GSE58812 (**Figure 3D**). Furthermore, patients with different risks (27 in the high-risk subgroup *v.s.* 34 in the low-risk subgroup) were assigned to two clusters in GSE37751 (**Figure 3E**). In GSE37751, patients in the high-risk group had shorter survival than those patients in the low-risk group (P = 0.029, **Figure 3F**). Also, the survival status was shown in **Figure 3G**, and the 1-, 3-, and 5-year AUC values were 0.636, 0.702, and 0.738 (**Figure 3H**). We additionally compared expressions of key genes between human breast cancer cells and a human mammary epithelial cell line MCF-10A using RT-qPCR assay. As shown, compared with MCF-10A, the expression levels of GSDMC, GZMB and IL18 were upregulated, while TP63 was found with lower expression level in breast cancer cells SK-BR-3, BT-549, MCF-7, and MDA-MB-231 (**Figures 3I–L**, P < 0.05).

### 3.4 Analysis of clinicopathological relevance and functional enrichment

**Figure 4:**
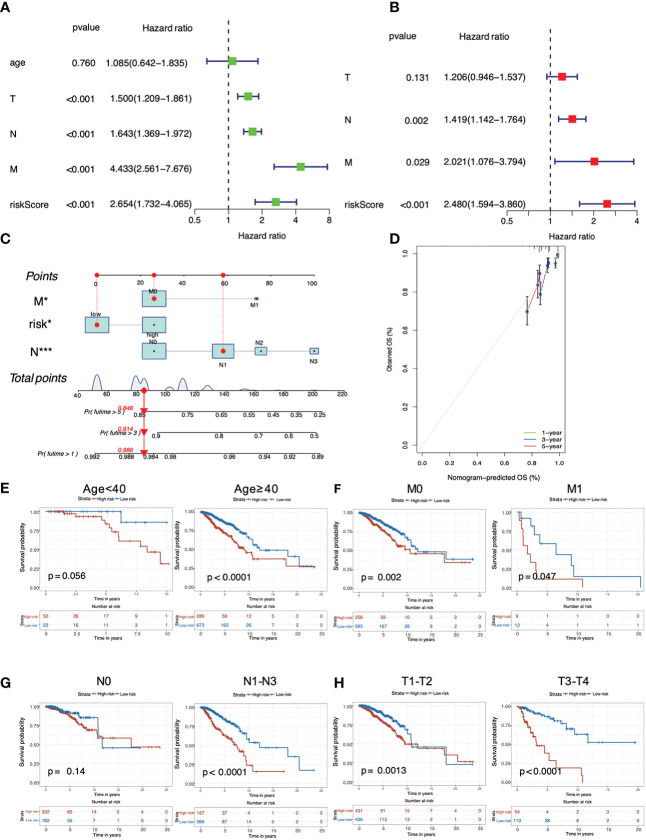
Analysis of predictive independency and clinicopathological relevance of the PRG signature in breast cancer. **(A)** Univariate Cox regression analysis; **(B)** Multivariate Cox regression analysis; **(C)** The nomogram for predicting OS probabilities for breast cancer patients with either high or low risk; **(D)** The 1-, 3- and 5-year nomogram calibration curves; Subgroup analysis of **(E)** age (< 40 years and ≥ 40 years), **(F)** M stage (M0 and M1), **(G)** N stage (N0 and N1-N3) and **(H)** T stage (T1-T2 and T3-T4).

Both univariate (**Figure 4A**) and multivariate (**Figure 4B**) Cox regression analyses proved that the PRG-based signature, as well as N stage and M stage, were independent predictors for poor prognosis of breast cancer patients (P < 0.05), demonstrating the robustness and accuracy of our method. The calibration curve for OS probability at 1, 3, and 5 years also suggested satisfactory consistency between the predicted and actual survival probabilities (C-index value=0.69, **Figures 4C, D**). Next, we continued to investigate the clinicopathological correlation of breast cancer with this PRG risk model in TCGA. As shown, the PRG signature in the high-risk group was significantly correlated with poor survival in older (≥ 40 years), M0 or M1, N1-N3, and T1-T2 or T3-T4 populations (P < 0.05; **Figures 4E–H**). Taken together, this PRG signature with high-risk score was possibly an independent prognostic marker linking to a poor clinical outcome for breast cancer patients.

**Figure 5:**
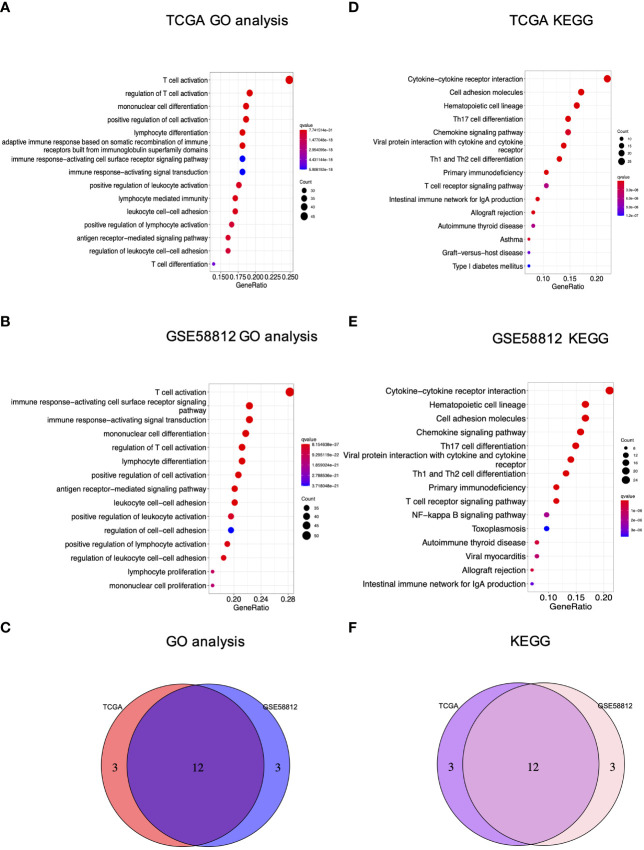
GO and KEGG analyses of the PRG signature in breast cancer. **(A)** GO analysis of the TCGA cohort; **(B)** GO analysis of GSE58812; **(C)** The Venn diagram of GO terms between the TCGA and GEO cohorts; **(D)** KEGG analysis of the TCGA cohort; **(E)** KEGG analysis of GSE58812; **(F)** The Venn diagram of KEGG terms between the TCGA and GEO cohorts.

In addition, enrichment analyses were carried out to reveal the molecular functions and underlying mechanisms associated with pyroptosis in breast cancer. For GO functional analysis, the terms existing in both the TCGA and GSE58812 cohorts included T cell activation, regulation of T cell activation, mononuclear cell differentiation, positive regulation of cell activation, lymphocyte differentiation, immune response minus;activating cell surface receptor signaling pathway, immune response−activating signal transduction, positive regulation of leukocyte activation, leukocyte cell−cell adhesion, positive regulation of lymphocyte activation, antigen receptor−mediated signaling pathway and regulation of leukocyte cell−cell adhesion (**Figures 5A–C**). For KEGG analysis, bubble charts demonstrated that the involved pathways co-existing in both cohorts were mainly associated with immunological modulation and cancer interference, including cytokine−cytokine receptor interaction, cell adhesion molecules, hematopoietic cell lineage, Th17 cell differentiation, chemokine signaling pathway, viral protein interaction with cytokine and cytokine receptor, Th1 and Th2 cell differentiation, primary immunodeficiency, T cell receptor signaling pathway, intestinal immune network for IgA production, allograft rejection, and autoimmune thyroid disease (**Figures 5D–F**).

### 3.5 Analysis of immunological status and TME characterization

**Figure 6:**
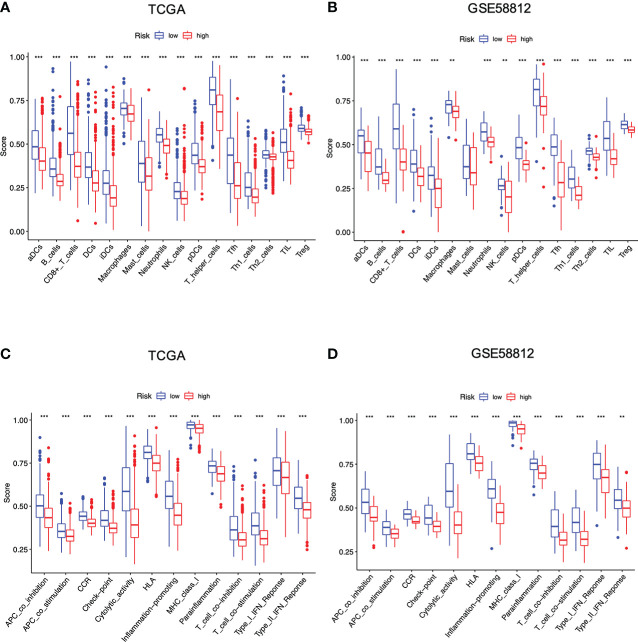
Differences of infiltrating immune cells and immune-related pathways between different risk groups based on the TCGA and GEO cohort. **(A, B)** Infiltrating immune cells between two risk groups; **(C, D)** Immune-related pathways between two risk groups (all **P < 0.01; ***P < 0.001).

On this basis, ssGSEA was then performed to evaluate the impact of this signature on immunological status, particularly immune cell types and functions. As shown, high-risk score led to lower levels of infiltrating immune cells, including active DCs (aDCs), B cells, CD8+T cells, dendritic cells (DCs), immature dendritic cells (iDCs), macrophages, neutrophils, natural killer (NK) cells, plasmacytoid DCs (pDCs), T helper cells, T follicular helper (Tfh) cells, Th1 cells, Th2 cells, tumor infiltrating lymphocyte (TIL), and regulatory cell (Treg) in two datasets (**Figures 6A, B**). In addition, the high-risk individuals presented lower activities in 13 immune-related pathways, including Antigen presenting cell (APC) co-inhibition, APC co-stimulation, C-C chemokine receptor (CCR), check-point, cytolytic activity, human leukocyte antigen (HLA), inflammation-promoting, major histocompatibility complex (MHC) class I, parainflammation, T cell co-inhibition, T cell co-stimulation, type I interferon (IFN) response, and type II interferon (IFNγ) response (**Figures 6C, D**). Overall, these data suggested that high-risk conditions were largely associated with impaired immune function in breast cancer, possibly resulting in unfavorable outcomes for those patients.

**Figure 7:**
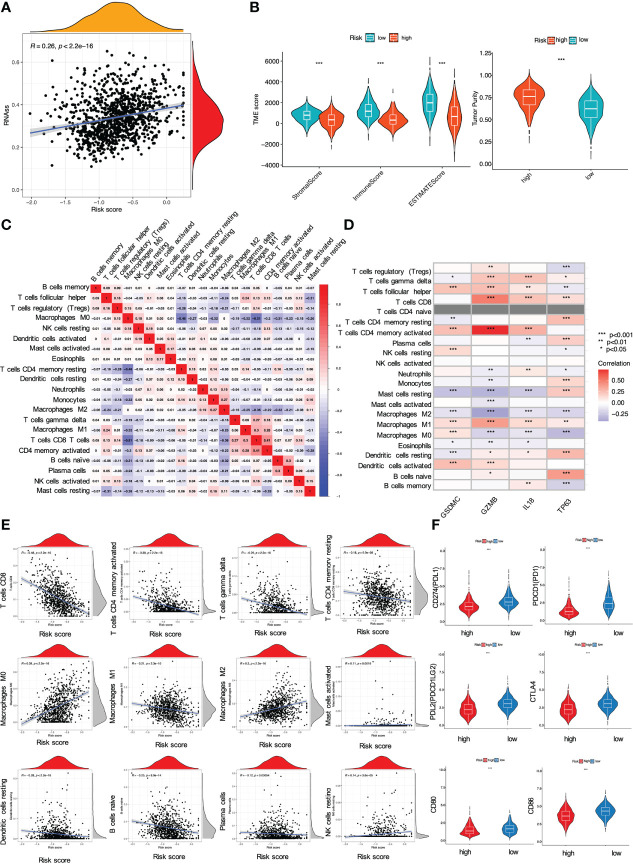
Impact of the risk signature on immune activities in TME. **(A)** Relationship between PRG score and cancer stem cells; **(B)** Analysis of PRGs related to immune/stromal/estimate score and tumor purity. The PRG signature correlated with **(C, D)** immune cell abundance, **(E)** immune cell types, and **(F)** checkpoints (all *P < 0.05; **P < 0.01; ***P < 0.001).

Moreover, we also investigated whether and how risk score affected immune activities in TME. Firstly, CSCs are the roots of breast cancer (31). In this investigation, breast cancer cells with higher PRG score also yielded a higher CSC index, indicating that it might be possible to eliminate CSCs through the use of pyroptosis-related therapies by targeting these 4 PRGs (P < 0.001, **Figure 7A**). Next, we estimated TME score of breast cancer samples with R package “ESTIMATE” in TCGA. The goal of this algorithm was to retrieve stromal, immune and estimate scores. In particular, both stromal and immune scores were positively correlated to infiltration of stromal and immune cells, while the estimate score (the sum of the stromal and immune scores) was a negative indicator of purity of tumor cells (32). Compared with the low-risk group, the high-risk group with lower immune/stromal/estimate score exhibited higher tumor purities (**Figure 7B**). Further analysis revealed that most immune cells exhibited significant correlations with the four PRGs GSDMC, GZMB, IL18, and TP63 (**Figures 7C, D**). Also, the risk signature had a direct bearing on immune cell types determined by CIBERSORT algorithm. In particular, the PRG score was negatively correlated with T cells CD4 memory activated, T cells CD8, T cells CD4 memory resting, T cells gamma delta, Macrophages M1, B cells naïve and Plasma cells, while positively related to Macrophages M0, Macrophages M2, Mast cells activated and NK cells resting (**Figure 7E**). We also compared the immune-checkpoint markers between two subgroups. As shown, patients in the low-risk group expressed significantly higher levels of PD-1, PD-L1, PDL-2, CD80, CD86, and CTLA-4, implying that immune checkpoint blockade (ICB) therapies might be effective for the low-risk patients (P < 0.001, **Figure 7F**).

### 3.6 Evaluation of drug sensitivity and genetic regulation

**Figure 8:**
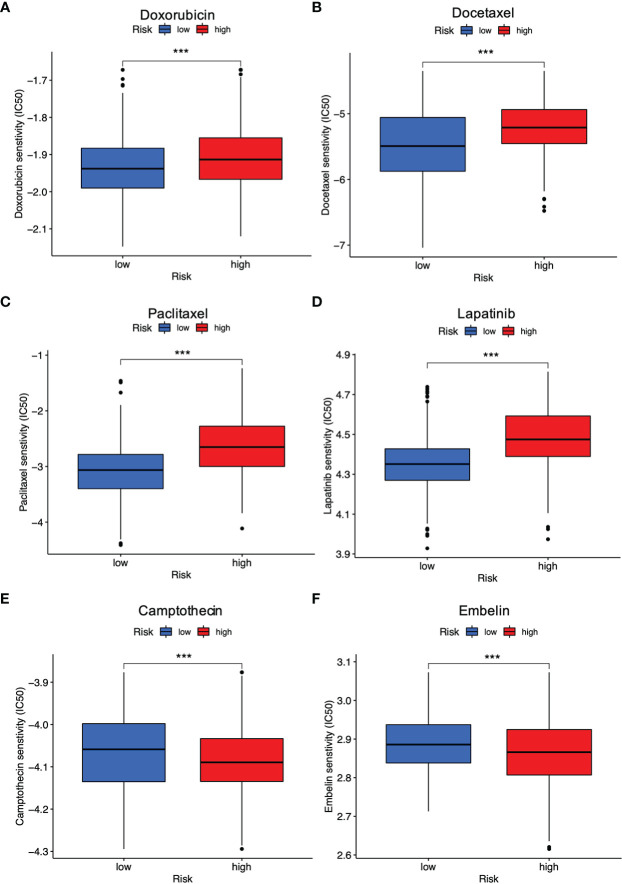
Evaluation of drug response between different risk groups. **(A)** Doxorubicin; **(B)** Docetaxel; **(C)** Paclitaxel; **(D)** Lapatinib; **(E)** Camptothecin; **(F)** Embelin (all ***P < 0.001).

The risk signature was then used to predict whether breast cancer patients could benefit from chemotherapeutic treatments or targeted therapies. Wilcoxon’s ranked-rank test was utilized to compare IC50 values between the high-risk and low-risk groups. As shown, the low-risk individuals with breast cancer had lower IC50 values for doxorubicin (**Figure 8A**), docetaxel (**Figure 8B**), paclitaxel (**Figure 8C**), lapatinib (**Figure 8D**), while IC50 values of drugs such as camptothecin (**Figure 8E**), embelin (**Figure 8F**) were obviously lower in breast cancer patients with high PRG risk (all P < 0.001). Overall, it was suggested that this signature was related to drug sensitivity and might provide guidance for treating breast cancer in the clinical setting.

In addition, we also investigated the genetic regulation of the four PRGs. An analysis of its molecular characteristics was conducted by searching the dataset of Breast Cancer (METABRIC, Nature 2012 & Nat Commun 2016) in cBioPortal. In particular, the OncoPrint tab summarized genomic alterations (including amplification, deletion, upregulation, and *etc.*) of TP63, IL18, GZMB, GSDMC were 8%, 0.2%, 5%, and 26%, respectively (**Figure S1A**). Of note, it was demonstrated that autophagy might play a crucial role in keeping intracellular homeostasis by regulating pyroptosis (33). By comparing 52 PRGs with 222 ARGs, 11 common genes TP53, NLRC4, BAK1, CASP1, CASP4, CASP8, BAX, CHMP4B, TP63, CASP3, and CHMP2B were identified, among which only TP63 exhibited further interactions with the established 4-gene signature (**Figure S1B**). According to both the TCGA and GEO cohorts, a decrease of TP63 level was an indicator for poor survival status for breast cancer patients (**Figure S1C–E**). In addition, a comparison between N0 and N1-N3 status of breast cancer patients demonstrated that cases with lymph-node metastasis had lower TP63 mRNA expression (P = 0.017, **Figure S1F**), which might be due to its hypomethylation (**Figure S1G**).

## 4 Discussion

It is always important to seek and decipher pyroptosis-associated targets in breast cancer. For instance, GSDME methylation at high frequency contributed to lymph node metastasis and a poor prognosis for breast cancer patients (34, 35). Pizato et al. revealed that omega-3 docosahexaenoic acid had robust facilitation of pyroptosis-mediated cell death in triple-negative breast cancer, improving the understanding of DHA as nutriment and adjuvant treatment against breast cancer (36). The study from Liang et al. pointed out that trichlorobendazole triggered GSDME-dependent pyroptosis of breast cancer cells and clarified the involved mechanism was associated with augment of ROS/JNK/Bax-mitochondrial signal, suggesting the potential therapeutic use of this drug for treating breast cancer patients with high GSDME expression (37). Cisplatin induced anti-breast cancer effects at least partly by activating MEG3/NLRP3/caspase-1/GSDMD pathway (38). In light of these findings, it is crucial to develop a PRG-based prognostic signature to clarify the significance of pyroptosis in breast cancer. Herein, the PRG-based risk model containing GSDMC, GZMB, IL18, and TP63 was established in the TCGA cohort, followed by further validation with the GEO cohorts and qRT-PCR assay. Retrospectively, these targets could affect cancer progression by regulating pyroptosis directly or indirectly. For instance, GSDMC was initially recognized as an oncogene in metastatic mouse melanoma. Pyroptosis could be induced by artificially truncated N-terminal GSDMC (GSDMC^NT^), but the upstream signaling of GSDMC still remained unclear. A recent study by Hou et al. supplied one possible explanation for such a research gap. They demonstrated that tumor hypoxia induced PD-L1 nuclear translocation, accompanied by activation of GSDMC expression and pyroptosis induction (39). In addition, GSDMC overexpression might be related to lung cancer progression and poor survival (40). It was also worth mentioning that TP63 was identified as a core target in various cancer diseases. Lin et al. revealed that increased expression of TP63 isoform TAp63 abrogated the invasive abilities of colon cancer cells HT-29 and SW-620 (41). In murine model, TP63 loss led to activation of MAPK-P-STAT3 (Ser727)-MMP15 axis, resulting in metastatic spread of head and neck squamous cell carcinoma (42). A decline in TP63 expression was related to shorter survival times of patients with breast cancer, bladder cancer, and lung cancer (43–45). However, the influence of TP63 on pyroptosis as a single gene was not well understood. Wang et al. identified TP63 as an autophagy-related gene in breast cancer (45). Given that pyroptosis could be controlled by autophagy (33), TP63 might be an indirect factor affecting pyroptosis. In addition, TP63 has two isoforms *i.e.* TAp63 and ΔNp63 (46), making it hard for clarifying its specified role in breast cancer. Breast cancer also contains several molecular subtypes, including HR+/ERBB2−, ERBB2+ as well as triple-negative (2). As such, it is not clear to identify which subtype of TP63 exerts a dominant role based on the present information retrieved from the bioinformatics database, and we will address this frustrating issue in our future study.

Since any single gene was insufficient to be an appropriate marker for pyroptosis, we investigated the 4 aforementioned PRGs as a whole for breast cancer prognostic and immunological evaluation in this study. Several studies also reported similar pyroptosis-related models for breast cancer. The study of Wu et al. retrieved 33 PRGs to evaluate their relation to breast cancer progression (47). On this basis, our investigation expanded the number to 52 PRGs for initiating our model, and 4 out of the 52 PRGs were selected as the appropriate candidates for constructing a pyroptosis-related risk signature. Of note, our study was partly consistent with the findings of Wu et al., revealing the supporting role of IL18 in breast cancer progression (47). In addition, Yu et al. incorporated 15 candidate genes i.e. NLRC4, IRF3, ANO6, GSDMC, TP53, FGF21, IL36B, DHX9, FOXO3, IL36G, IL18, GJA1, MST1, GZMB and GBP1 for the development of a PRG model related to breast cancer (48). Although several of these single hub genes were indirectly related to pyroptosis, their combination was demonstrated to be an accurate predictor of breast cancer survival. Compared to the study of Yu et al., our predictive signature had fewer genes, which might save costs for the development of a corresponding diagnostic kit in future clinical applications (48). Beyond the above findings, we additionally revealed that TP63 was the common gene to associate pyroptosis with autophagy. The decreased expression of TP63 might have potential relation to hypomethylation, and predicted a poor OS rate for breast cancer patients. Since autophagy contributed to intracellular homeostasis by modulating pyroptosis (33), it is interesting to investigate the role of methylation-induced TP63 reduction in breast cancer and whether the involved mechanisms will be related to autophagic regulation by interfering with pyroptosis in the next study.

Our study further indicated that the obtained signature was highly relevant to immune response based on a combined analysis of GO, KEGG, ssGSEA and CIBERSORT, indicating the profound implication of pyroptosis in tumor immunity. In particular, GO and KEGG analysis revealed the low-risk group was featured by enhancement of T cell function and regulation of cytokine-cytokine receptor interaction. Then, ssGSEA and CIBERSORT methods were to analyze the association between the PRG signature and immune cell infiltration. According to the results of ssGSEA, the low risk led to greater quantities of immune cells, including B cells, T cells, dendritic cells, macrophages and so on. These results were consistent with the findings of CIBERSORT algorithm, showing that the low risk was correlated with subtypes of immune-stimulating cells, such as activated T cells, M1 macrophages, and dendritic cells, as well as B cells. In most cases, these cells might activate the immune system, leading to a positive prognosis of cancer diseases (49). Herein, it was confirmed by our results showing that T cells, M1 macrophages, dendritic cells and B cells were enriched in the low-risk group with a favorable prognosis in breast cancer. Furthermore, blockage of the immune checkpoint has become a trend in immunotherapy for breast cancer. Breast cancer patients have greatly benefited from immune checkpoint inhibitors by targeting PD-1 and PD-L1. A clinical trial revealed that atezolizumab, targeting PD-L1 protein, combined with nab-paclitaxel could be used to treat patients with metastatic triple-negative breast cancer (50). Also, KEYNOTE-012 and KEYNOTE-086 trials indicated that pembrolizumab was a PD-1 targeted immune checkpoint blocker for TNBC (51). In our study, it was shown that the low-risk patients had significantly higher levels of PD-1, PD-L1, PD-L2, CD80, CD86, and CTLA-4 than the high-risk patients, suggesting this low-risk subpopulation of breast cancer patients might benefit more from immune checkpoint blockade therapy. The above data suggested that the established PRG-related signature was tightly related to immune activation and tolerance.

Clinical outcomes and efficacy are hampered by acquired drug resistance in cancer diseases. In this study, it was found that the low-risk breast cancer patients were more sensitive to doxorubicin, docetaxel, paclitaxel, as well as lapatinib, whereas the high-risk populations were more responsive to camptothecin and embelin. Among the above therapeutic agents, doxorubicin, docetaxel and paclitaxel are common chemotherapeutic agents for breast cancer (52). There was evidence showing that paclitaxel could induce pyroptosis by activating Caspase-3/GSDME (53). Lapatinib is one of tyrosine kinase inhibitors targeting epidermal growth factor receptor (EGFR/ErbB1) as well as HER2/ErbB2 specifically for treating HER2+ subtypes of breast cancer (54). Moreover, camptothecin targets the nuclear enzyme topoisomerase I (TOP1) to treat endocrine-resistant breast cancer (55), and embelin is capable of inducing apoptosis in MCF-7 breast cancer cells (56). Individuals with breast cancer responded differently to these therapeutic agents on the basis of our PRG-based signature, and such prediction is aimed to decide which patients would benefit most from certain treatments. In other words, we hope to be capable of predicting novel drugs, identifying new therapeutic targets, and providing individualized treatment to breast cancer patients with such a model in the future.

## 5 Conclusion

Taken together, our study identified a 4-gene PRG signature tightly associated with survival status and immunological landscape, providing basic guidance for immunotherapy and individualized treatment in breast cancer.

## Data availability statement

The datasets presented in this study can be found in online repositories. The names of the repository/repositories and accession number(s) can be found in the article/**Supplementary Material**.

## Author contributions

YZ and NW designed the study and wrote the first draft. ZW and CP revised the manuscript. YZ and FP conducted the bioinformatic analysis. YZ and XL conducted biological experiments for further validation. XL, XW and BY participated in data interpretation. XT, ZX and LR contributed to data collection and discussion. This manuscript has been approved by all authors for publication.
