# Supplementary material for: A pyroptosis-related gene signature for prognostic and immunological evaluation in breast cancer
Source: Front Oncol. 2022 Dec 23;12:964508. doi: 10.3389/fonc.2022.964508 (PMC10020702; doi:10.3389/fonc.2022.964508)
Supplement: Supplementary file 1 [file DataSheet_1.docx]

Supplementary Materials

## Supplementary figure legend:


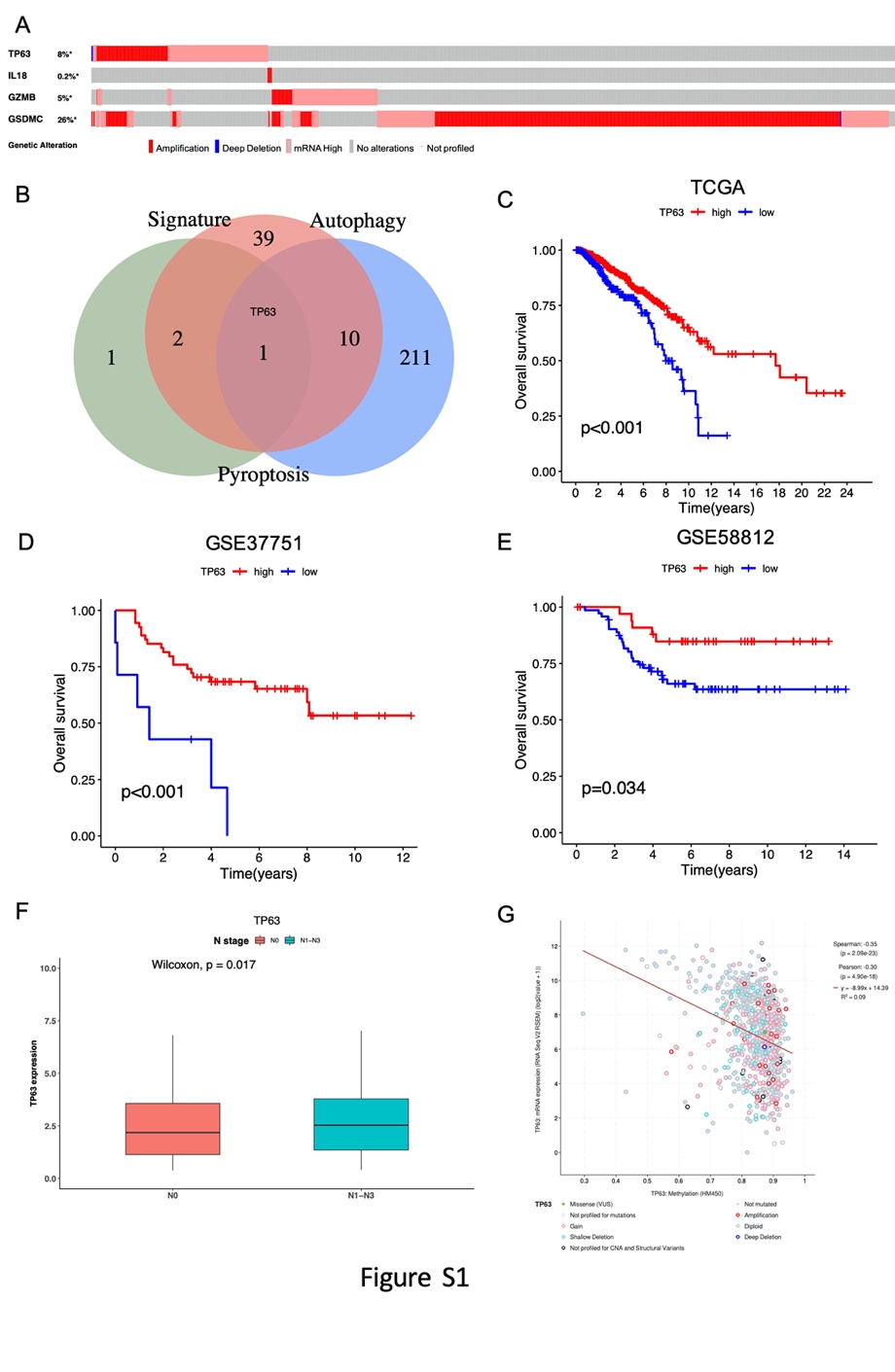


**Figure S1. TP63 was the common gene to associate pyroptosis with autophagy. (A)** Genomic alterations (including amplification, deletion, upregulation and others) of TP63, IL18, GZMB and GSDMC; **(B)** The venn diagram revealed that TP63 might be one of the key genes linking pyroptosis to autophagy; **(C-E)** TP63 downregulation correlated with poor survival status in breast cancer; **(F)** lymph-node metastasis, and **(F)** methylation status of TP63.

## Supplementary Tables:

**Table S1. Information of PRGs**

| **Gene** | **Full-names** |
| --- | --- |
| AIM2 | Absent In Melanoma 2 |
| BAK1 | BCL2 Antagonist/Killer 1 |
| BAX | BCL2 Associated X |
| CASP1 | Caspase 1 |
| CASP3 | Caspase 3 |
| CASP4 | Caspase 4 |
| CASP5 | Caspase 5 |
| CASP6 | Caspase 6 |
| CASP8 | Caspase 8 |
| CASP9 | Caspase 9 |
| ELANE | Elastase, Neutrophil Expressed |
| CHMP2A | Charged Multivesicular Body Protein 2A |
| CHMP2B | Charged Multivesicular Body Protein 2B |
| CHMP3 | Charged Multivesicular Body Protein 3 |
| CHMP4A | Charged Multivesicular Body Protein 4A |
| CHMP4B | Charged Multivesicular Body Protein 4B |
| CHMP4C | Charged Multivesicular Body Protein 4C |
| CHMP6 | Charged Multivesicular Body Protein 6 |
| CHMP7 | Charged Multivesicular Body Protein 7 |
| CYCS | Cytochrome C |
| GPX4 | Glutathione Peroxidase 4 |
| GSDMA | Gasdermin A |
| GSDMB | Gasdermin B |
| GSDMC | Gasdermin C |
| GSDMD | Gasdermin D |
| GSDME | Gasdermin E |
| GZMA | Granzyme A |
| GZMB | Granzyme B |
| HMGB1 | High Mobility Group Box 1 |
| IL1A | Interleukin 1 Alpha |
| IL18 | Interleukin 18 |
| IL1B | Interleukin 1 beta |
| IL6 | Interleukin 6 |
| IRF1 | Interferon Regulatory Factor 1 |
| IRF2 | Interferon Regulatory Factor 2 |
| NLRC4 | NLR family CARD Domain Containing 4 |
| NLRP1 | NLR Family Pyrin Domain Containing 1 |
| NLRP2 | NLR Family Pyrin Domain Containing 2 |
| NLRP3 | NLR Family Pyrin Domain Containing g 3 |
| NLRP6 | NLR Family Pyrin Domain Containing 6 |
| NLRP7 | NLR Family Pyrin Domain Containing 7 |
| NOD1 | Nucleotide Binding Oligomerization Domain Containing 1 |
| NOD2 | Nucleotide Binding Oligomerization Domain Containing 2 |
| PJVK | Pejvakin |
| PLCG1 | Phospholipase C Gamma 1 |
| PRKACA | Protein Kinase CAMP-Activated Catalytic Subunit Alpha |
| PYCARD | PYD And CARD Domain Containing |
| SCAF11 | SR-Related CTD Associated Factor 11 |
| TIRAP | TIR Domain Containing Adaptor Protein |
| TNF | Tumor Necrosis Factor |
| TP53 | Tumor Protein P53 |
| TP63 | Tumor Protein P63 |

**Table S2. PCR primer sequences were used in the study.**

| **Primer name** |  | **Primer sequences** |
| --- | --- | --- |
| GSDMC | Forward primer | GAGGGGACAACCTGTACGTG |
|  | Reverse primer | TCTGAAGAGTCAGCGCCTTC |
| GZMB | Forward primer | CCCTGGGAAAACACTCACACA |
|  | Reverse primer | GCACAACTCAATGGTACTGTCG |
| IL18 | Forward primer | TCTTCATTGACCAAGGAAATCGG |
|  | Reverse primer | TCCGGGGTGCATTATCTCTAC |
| TP63 | Forward primer | GTCATTTGATTCGAGTAGAGGGG |
|  | Reverse primer | CTGGGGTGGCTCATAAGGT |
| β-actin | Forward primer | CCAACCGCGAGAAGATGA |
|  | Reverse primer | CCAGAGGCGTACAGGGATAG |

**Table S3. Information of ARGs**

| **Gene** | **Full-names** |
| --- | --- |
| AMBRA1 | autophagy/beclin-1 regulator 1 |
| APOL1 | apolipoprotein L, 1 |
| ARNT | aryl hydrocarbon receptor nuclear translocator |
| ARSA | arylsulfatase A |
| ARSB | arylsulfatase B |
| ATF4 | activating transcription factor 4 (tax-responsive enhancer element B67) |
| ATF6 | activating transcription factor 6 |
| ATG10 | ATG10 autophagy related 10 homolog (S. cerevisiae) |
| ATG12 | ATG12 autophagy related 12 homolog (S. cerevisiae) |
| ATG16L1 | ATG16 autophagy related 16-like 1 (S. cerevisiae) |
| ATG16L2 | ATG16 autophagy related 16-like 2 (S. cerevisiae) |
| ATG2A | ATG2 autophagy related 2 homolog A (S. cerevisiae) |
| ATG2B | ATG2 autophagy related 2 homolog B (S. cerevisiae) |
| ATG3 | ATG3 autophagy related 3 homolog (S. cerevisiae) |
| ATG4A | ATG4 autophagy related 4 homolog A (S. cerevisiae) |
| ATG4B | ATG4 autophagy related 4 homolog B (S. cerevisiae) |
| ATG4C | ATG4 autophagy related 4 homolog C (S. cerevisiae) |
| ATG4D | ATG4 autophagy related 4 homolog D (S. cerevisiae) |
| ATG5 | ATG5 autophagy related 5 homolog (S. cerevisiae) |
| ATG7 | ATG7 autophagy related 7 homolog (S. cerevisiae) |
| ATG9A | ATG9 autophagy related 9 homolog A (S. cerevisiae) |
| ATG9B | ATG9 autophagy related 9 homolog B (S. cerevisiae) |
| ATIC | 5-aminoimidazole-4-carboxamide ribonucleotide formyltransferase/IMP cyclohydrolase |
| BAG1 | BCL2-associated athanogene |
| BAG3 | BCL2-associated athanogene 3 |
| BAK1 | BCL2-antagonist/killer 1 |
| BAX | BCL2-associated X protein |
| BCL2 | B-cell CLL/lymphoma 2 |
| BCL2L1 | BCL2-like 1 |
| BECN1 | beclin 1, autophagy related |
| BID | BH3 interacting domain death agonist |
| BIRC5 | baculoviral IAP repeat-containing 5 |
| BIRC6 | baculoviral IAP repeat-containing 6 |
| BNIP1 | BCL2/adenovirus E1B 19kDa interacting protein 1 |
| BNIP3 | BCL2/adenovirus E1B 19kDa interacting protein 3 |
| BNIP3L | BCL2/adenovirus E1B 19kDa interacting protein 3-like |
| C12orf44 | chromosome 12 open reading frame 44 |
| C17orf88 | chromosome 17 open reading frame 88 |
| CALCOCO2 | calcium binding and coiled-coil domain 2 |
| CAMKK2 | calcium/calmodulin-dependent protein kinase kinase 2, beta |
| CANX | calnexin |
| CAPN1 | calpain 1, (mu/I) large subunit |
| CAPN10 | calpain 10 |
| CAPN2 | calpain 2, (m/II) large subunit |
| CAPNS1 | calpain, small subunit 1 |
| CASP1 | caspase 1, apoptosis-related cysteine peptidase (interleukin 1, beta, convertase) |
| CASP3 | caspase 3, apoptosis-related cysteine peptidase |
| CASP4 | caspase 4, apoptosis-related cysteine peptidase |
| CASP8 | caspase 8, apoptosis-related cysteine peptidase |
| CCL2 | chemokine (C-C motif) ligand 2 |
| CCR2 | chemokine (C-C motif) receptor 2 |
| CD46 | CD46 molecule, complement regulatory protein |
| CDKN1A | cyclin-dependent kinase inhibitor 1A (p21, Cip1) |
| CDKN1B | cyclin-dependent kinase inhibitor 1B (p27, Kip1) |
| CDKN2A | cyclin-dependent kinase inhibitor 2A (melanoma, p16, inhibits CDK4) |
| CFLAR | CASP8 and FADD-like apoptosis regulator |
| CHMP2B | chromatin modifying protein 2B |
| CHMP4B | chromatin modifying protein 4B |
| CLN3 | ceroid-lipofuscinosis, neuronal 3 |
| CTSB | cathepsin B |
| CTSD | cathepsin D |
| CTSL1 | cathepsin L1 |
| CX3CL1 | chemokine (C-X3-C motif) ligand 1 |
| CXCR4 | chemokine (C-X-C motif) receptor 4 |
| DAPK1 | death-associated protein kinase 1 |
| DAPK2 | death-associated protein kinase 2 |
| DDIT3 | DNA-damage-inducible transcript 3 |
| DIRAS3 | DIRAS family, GTP-binding RAS-like 3 |
| DLC1 | deleted in liver cancer 1 |
| DNAJB1 | DnaJ (Hsp40) homolog, subfamily B, member 1 |
| DNAJB9 | DnaJ (Hsp40) homolog, subfamily B, member 9 |
| DRAM1 | DNA-damage regulated autophagy modulator 1 |
| EDEM1 | ER degradation enhancer, mannosidase alpha-like 1 |
| EEF2 | eukaryotic translation elongation factor 2 |
| EEF2K | eukaryotic elongation factor-2 kinase |
| EGFR | epidermal growth factor receptor (erythroblastic leukemia viral (v-erb-b) oncogene homolog, avian) |
| EIF2AK2 | eukaryotic translation initiation factor 2-alpha kinase 2 |
| EIF2AK3 | eukaryotic translation initiation factor 2-alpha kinase 3 |
| EIF2S1 | eukaryotic translation initiation factor 2, subunit 1 alpha, 35kDa |
| EIF4EBP1 | eukaryotic translation initiation factor 4E binding protein 1 |
| EIF4G1 | eukaryotic translation initiation factor 4 gamma, 1 |
| ERBB2 | v-erb-b2 erythroblastic leukemia viral oncogene homolog 2, neuro/glioblastoma derived oncogene homolog (avian) |
| ERN1 | endoplasmic reticulum to nucleus signaling 1 |
| ERO1L | ERO1-like (S. cerevisiae) |
| FADD | Fas (TNFRSF6)-associated via death domain |
| FAM48A | family with sequence similarity 48, member A |
| FAS | Fas (TNF receptor superfamily, member 6) |
| FKBP1A | FK506 binding protein 1A, 12kDa |
| FKBP1B | FK506 binding protein 1B, 12.6 kDa |
| FOS | FBJ murine osteosarcoma viral oncogene homolog |
| FOXO1 | forkhead box O1 |
| FOXO3 | forkhead box O3 |
| GAA | glucosidase, alpha; acid |
| GABARAP | GABA(A) receptor-associated protein |
| GABARAPL1 | GABA(A) receptor-associated protein like 1 |
| GABARAPL2 | GABA(A) receptor-associated protein-like 2 |
| GAPDH | glyceraldehyde-3-phosphate dehydrogenase |
| GNAI3 | guanine nucleotide binding protein (G protein), alpha inhibiting activity polypeptide 3 |
| GNB2L1 | guanine nucleotide binding protein (G protein), beta polypeptide 2-like 1 |
| GOPC | golgi-associated PDZ and coiled-coil motif containing |
| GRID1 | glutamate receptor, ionotropic, delta 1 |
| GRID2 | glutamate receptor, ionotropic, delta 2 |
| HDAC1 | histone deacetylase 1 |
| HDAC6 | histone deacetylase 6 |
| HGS | hepatocyte growth factor-regulated tyrosine kinase substrate |
| HIF1A | hypoxia inducible factor 1, alpha subunit (basic helix-loop-helix transcription factor) |
| HSP90AB1 | heat shock protein 90kDa alpha (cytosolic), class B member 1 |
| HSPA5 | heat shock 70kDa protein 5 (glucose-regulated protein, 78kDa) |
| HSPA8 | heat shock 70kDa protein 8 |
| HSPB8 | heat shock 22kDa protein 8 |
| IFNG | interferon, gamma |
| IKBKB | inhibitor of kappa light polypeptide gene enhancer in B-cells, kinase beta |
| IKBKE | inhibitor of kappa light polypeptide gene enhancer in B-cells, kinase epsilon |
| IL24 | interleukin 24 |
| IRGM | immunity-related GTPase family, M |
| ITGA3 | integrin, alpha 3 (antigen CD49C, alpha 3 subunit of VLA-3 receptor) |
| ITGA6 | integrin, alpha 6 |
| ITGB1 | integrin, beta 1 (fibronectin receptor, beta polypeptide, antigen CD29 includes MDF2, MSK12) |
| ITGB4 | integrin, beta 4 |
| ITPR1 | inositol 1,4,5-triphosphate receptor, type 1 |
| KIAA0226 | KIAA0226 |
| KIAA0652 | KIAA0652 |
| KIAA0831 | KIAA0831 |
| KIF5B | kinesin family member 5B |
| KLHL24 | kelch-like 24 (Drosophila) |
| LAMP1 | lysosomal-associated membrane protein 1 |
| LAMP2 | lysosomal-associated membrane protein 2 |
| MAP1LC3A | microtubule-associated protein 1 light chain 3 alpha |
| MAP1LC3B | microtubule-associated protein 1 light chain 3 beta |
| MAP1LC3C | microtubule-associated protein 1 light chain 3 gamma |
| MAP2K7 | mitogen-activated protein kinase kinase 7 |
| MAPK1 | mitogen-activated protein kinase 1 |
| MAPK3 | mitogen-activated protein kinase 3 |
| MAPK8 | mitogen-activated protein kinase 8 |
| MAPK8IP1 | mitogen-activated protein kinase 8 interacting protein 1 |
| MAPK9 | mitogen-activated protein kinase 9 |
| MBTPS2 | membrane-bound transcription factor peptidase, site 2 |
| MLST8 | MTOR associated protein, LST8 homolog (S. cerevisiae) |
| MTMR14 | myotubularin related protein 14 |
| MTOR | mechanistic target of rapamycin (serine/threonine kinase) |
| MYC | v-myc myelocytomatosis viral oncogene homolog (avian) |
| NAF1 | nuclear assembly factor 1 homolog (S. cerevisiae) |
| NAMPT | nicotinamide phosphoribosyltransferase |
| NBR1 | neighbor of BRCA1 gene 1 |
| NCKAP1 | NCK-associated protein 1 |
| NFE2L2 | nuclear factor (erythroid-derived 2)-like 2 |
| NFKB1 | nuclear factor of kappa light polypeptide gene enhancer in B-cells 1 |
| NKX2-3 | NK2 transcription factor related, locus 3 (Drosophila) |
| NLRC4 | NLR family, CARD domain containing 4 |
| NPC1 | Niemann-Pick disease, type C1 |
| NRG1 | neuregulin 1 |
| NRG2 | neuregulin 2 |
| NRG3 | neuregulin 3 |
| P4HB | prolyl 4-hydroxylase, beta polypeptide |
| PARK2 | Parkinson disease (autosomal recessive, juvenile) 2, parkin |
| PARP1 | poly (ADP-ribose) polymerase 1 |
| PEA15 | phosphoprotein enriched in astrocytes 15 |
| PELP1 | proline, glutamate and leucine rich protein 1 |
| PEX14 | peroxisomal biogenesis factor 14 |
| PEX3 | peroxisomal biogenesis factor 3 |
| PIK3C3 | phosphoinositide-3-kinase, class 3 |
| PIK3R4 | phosphoinositide-3-kinase, regulatory subunit 4 |
| PINK1 | PTEN induced putative kinase 1 |
| PPP1R15A | protein phosphatase 1, regulatory (inhibitor) subunit 15A |
| PRKAB1 | protein kinase, AMP-activated, beta 1 non-catalytic subunit |
| PRKAR1A | protein kinase, cAMP-dependent, regulatory, type I, alpha (tissue specific extinguisher 1) |
| PRKCD | protein kinase C, delta |
| PRKCQ | protein kinase C, theta |
| PTEN | phosphatase and tensin homolog |
| PTK6 | PTK6 protein tyrosine kinase 6 |
| RAB11A | RAB11A, member RAS oncogene family |
| RAB1A | RAB1A, member RAS oncogene family |
| RAB24 | RAB24, member RAS oncogene family |
| RAB33B | RAB33B, member RAS oncogene family |
| RAB5A | RAB5A, member RAS oncogene family |
| RAB7A | RAB7A, member RAS oncogene family |
| RAC1 | ras-related C3 botulinum toxin substrate 1 (rho family, small GTP binding protein Rac1) |
| RAF1 | v-raf-1 murine leukemia viral oncogene homolog 1 |
| RB1 | retinoblastoma 1 |
| RB1CC1 | RB1-inducible coiled-coil 1 |
| RELA | v-rel reticuloendotheliosis viral oncogene homolog A (avian) |
| RGS19 | regulator of G-protein signaling 19 |
| RHEB | Ras homolog enriched in brain |
| RPS6KB1 | ribosomal protein S6 kinase, 70kDa, polypeptide 1 |
| RPTOR | regulatory associated protein of MTOR, complex 1 |
| SAR1A | SAR1 homolog A (S. cerevisiae) |
| SERPINA1 | serpin peptidase inhibitor, clade A (alpha-1 antiproteinase, antitrypsin), member 1 |
| SESN2 | sestrin 2 |
| SH3GLB1 | SH3-domain GRB2-like endophilin B1 |
| SIRT1 | sirtuin (silent mating type information regulation 2 homolog) 1 (S. cerevisiae) |
| SIRT2 | sirtuin (silent mating type information regulation 2 homolog) 2 (S. cerevisiae) |
| SPHK1 | sphingosine kinase 1 |
| SPNS1 | spinster homolog 1 (Drosophila) |
| SQSTM1 | sequestosome 1 |
| ST13 | suppression of tumorigenicity 13 (colon carcinoma) (Hsp70 interacting protein) |
| STK11 | serine/threonine kinase 11 |
| TBK1 | TANK-binding kinase 1 |
| TM9SF1 | transmembrane 9 superfamily member 1 |
| TMEM49 | transmembrane protein 49 |
| TMEM74 | transmembrane protein 74 |
| TNFSF10 | tumor necrosis factor (ligand) superfamily, member 10 |
| TP53 | tumor protein p53 |
| TP53INP2 | tumor protein p53 inducible nuclear protein 2 |
| TP63 | tumor protein p63 |
| TP73 | tumor protein p73 |
| TSC1 | tuberous sclerosis 1 |
| TSC2 | tuberous sclerosis 2 |
| TUSC1 | tumor suppressor candidate 1 |
| ULK1 | unc-51-like kinase 1 (C. elegans) |
| ULK2 | unc-51-like kinase 2 (C. elegans) |
| ULK3 | unc-51-like kinase 3 (C. elegans) |
| USP10 | ubiquitin specific peptidase 10 |
| UVRAG | UV radiation resistance associated gene |
| VAMP3 | vesicle-associated membrane protein 3 (cellubrevin) |
| VAMP7 | vesicle-associated membrane protein 7 |
| VEGFA | vascular endothelial growth factor A |
| WDFY3 | WD repeat and FYVE domain containing 3 |
| WDR45 | WD repeat domain 45 |
| WDR45L | WDR45-like |
| WIPI1 | WD repeat domain, phosphoinositide interacting 1 |
| WIPI2 | WD repeat domain, phosphoinositide interacting 2 |
